# In Vivo and In Silico Evaluation of Analgesic and Hypoglycemic Activities of Methanolic Extract of *Codariocalyx gyroids*

**DOI:** 10.1002/fsn3.70485

**Published:** 2025-06-29

**Authors:** Nilufar Sultana, Mahmudul Hasan, Ishmam Ibnul Arabi, Zobayed Islam, Joana Julhash, Umme Hani, Anika Sikder, Most Afroza Khanam, Abdullah Ripon

**Affiliations:** ^1^ Department of Pharmacy School of Engineering, Science and Technology Manarat International University Dhaka Bangladesh; ^2^ Department of Chemistry Faculty of Science, University of Chittagong Chattogram Bangladesh; ^3^ Department of Natural Science Port City International University Chattogram Bangladesh; ^4^ Department of Pharmacy, Faculty of Biological Science University of Chittagong Chattogram Bangladesh

**Keywords:** analgesic, *Codariocalyx gyroids*, hypoglycemic, methanolic extract, molecular docking

## Abstract

*Codariocalyx gyroides* (Roxb. ex Link) Hassk has long been recognized in traditional medicine for its diverse pharmacological attributes. However, its potential hypoglycemic and analgesic properties remain insufficiently elucidated. This study aimed to comprehensively evaluate the in vivo hypoglycemic and analgesic activities of the methanolic extract of *Codariocalyx gyroides* (MECG), supported by in silico molecular docking analyses to elucidate possible mechanisms of action. The acetic acid‐induced writhing assay investigated the analgesic activity in Swiss albino mice. MECG demonstrated dose‐dependent efficacy, significantly reducing writhing counts at 200 mg/kg (45.66) and 400 mg/kg (55.11). The hypoglycemic potential of MECG was assessed in a Streptozotocin (STZ)‐induced diabetic murine model, where a 200 mg/kg dose elicited a substantial reduction in plasma glucose levels. Complementary in silico analysis identified stigmasterol and lanosterol as key bioactive compounds. Stigmasterol exhibited a superior docking score (−7.9 kcal/mol) against cyclooxygenase‐2 (COX‐2) relative to diclofenac (−7.2 kcal/mol), suggesting a mechanistic basis for the observed analgesic effects. Lanosterol demonstrated enhanced affinity (−10.3 kcal/mol) for the sulfonylurea receptor, outperforming the standard hypoglycemic agent Glibenclamide (−9.2 kcal/mol). These findings underscore the therapeutic potential of MECG as a dual‐acting pharmacological agent, warranting further investigation to validate its clinical applicability in managing pain and diabetes.

## Graphical abstract

*Codariocalyx gyroides* (Roxb. ex Link) Hassk has been traditionally valued for its medicinal properties, yet its hypoglycemic and analgesic effects remain underexplored. This study demonstrated that the methanolic extract of *Codariocalyx gyroides* (MECG) exhibited dose‐dependent analgesic efficacy in Swiss albino mice and significantly reduced plasma glucose levels in a Streptozotocin‐induced diabetic model. In silico molecular docking identified stigmasterol and lanosterol as key bioactives, providing mechanistic insights into MECG's dual pharmacological potential, highlighting its promise for pain and diabetes management.

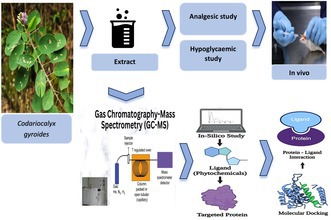

**Abbreviations**
ADME: administration, distribution, metabolism and excretion
COX: cyclooxygenase
MECG: methanolic extract of *Codariocalyx gyroides*
NSAID: non‐steroidal anti‐inflammatory drugs
PDB: Protein Data Bank
RO5: Lipinski's rule of five
SPSS: statistical package for the social sciences
STZ: streptozotocin
SUR1: sulfonylurea receptor‐1
T2D: Type II diabetes

## 1 Introduction

The utilization of medicinal plants for the prevention and treatment of various ailments has been an enduring practice throughout human history (Nongmaithem et al. 2018). Increasing demand for herbal therapeutic approaches has led to extensive exploration of medicinal species within traditional frameworks such as Ayurveda. Numerous biologically active secondary metabolites have been isolated from these plants, providing promising leads for the development of safer and more effective remedies (Mengle‐Gaw and Schwartz 2002; Muhammad and Fatima 2015; Teoh 2016). Currently, more than 4 billion people globally use herbal medicines for health care, indicating their widespread relevance.

Medicinal plants represent a rich source of pharmacologically active compounds and serve as important candidates for novel drug discovery. As such, they are now central to research initiatives aimed at scientifically validating traditional knowledge. The focus of these investigations is to develop economically viable therapeutic agents with minimal adverse effects (Fan et al. 2014; Kumar et al. 2023; Rodríguez‐Negrete et al. 2024; Sultana et al. 2013).

The pharmacological relevance of indigenous medicinal plants has supported the development of alternative herbal, Ayurvedic, and traditional Chinese medicines for managing a wide range of disorders. In our continued efforts to explore the therapeutic efficacy of native flora, our group has systematically studied several medicinal plants. Previous work has highlighted the pharmacodynamic properties and bioactive constituents of various plant extracts, supporting their traditional uses (George et al. 2021; Oliveros‐Díaz et al. 2021).

Pain is a complex sensory and emotional experience that serves a protective function (Roth‐Isigkeit et al. 2005). While NSAIDs—particularly non‐selective COX inhibitors—are widely used for pain relief, their prolonged use is associated with gastrointestinal, hepatic, renal, cardiovascular, and hematologic complications (Singh et al. 2018; Takeda et al. 2024). Even COX‐2 selective inhibitors pose cardiovascular risks over long‐term use. Consequently, medicinal plants are being investigated as safer analgesic options due to their diverse phytochemical content and comparatively fewer side effects (“Systematic Review of the Use of Phytochemicals for Management of Pain in Cancer Therapy, n.d.”).

Similarly, managing type 2 diabetes presents challenges when standard therapies are ineffective or cause adverse effects, including hypoglycemia, weight gain, and gastrointestinal distress (Hoda et al. 2019; Leone et al. 2022). Agents such as metformin may cause lactic acidosis; sulfonylureas are linked to cholestatic jaundice; and pioglitazone may lead to pulmonary edema (Panhwar et al. 2018). In contrast, herbal compounds have shown promising hypoglycemic activity with fewer side effects (Teoh and Das 2018; Eastern New Mexico University Portales NM USA et al. 2024).

This study aims to evaluate the analgesic and hypoglycemic properties of the methanolic extract of *Codariocalyx gyroides* (MECG) using in vivo and in silico approaches. The investigation focuses on two molecular targets—cyclooxygenase‐2 (COX‐2) and sulfonylurea receptor‐1 (SUR‐1)—to elucidate the potential of MECG as a dual‐action natural therapeutic agent.

## 2 Methods and Materials

### 2.1 Collection and Identification of Plant

The entire plant was collected from Khadimnagar, Sylhet, and subsequently identified at the Bangladesh National Herbarium, located in Mirpur, Dhaka‐1216. A voucher specimen, cataloged under the identification number DACB87278, has been deposited at the herbarium for future reference.

### 2.2 Drying and Grinding

The whole plant was meticulously washed and air‐dried under direct sunlight for 2–3 days. Once dried, the plant material was ground into a coarse powder using a grinder. The resulting powder was then stored in an airtight container to maintain its integrity for subsequent analysis.

### 2.3 Crude Methanolic Extract Preparation

Approximately 620 g of the crushed plant material were placed in a clean glass container, to which 2.5 L of methanol was added for maceration. The container was sealed, and the mixture was agitated for 15 days, with occasional shaking and stirring during storage. After 15 days, the entire mixture was coarsely filtered through a clean, white cotton cloth, followed by filtration using Whatman filter paper. The resulting filtrate (methanolic extract) was then concentrated using a rotary evaporator until completely dry, yielding a greenish‐black residue, which was designated as the crude methanol extract.

### 2.4 Drugs and Chemicals

Analytical‐grade solvents, including methanol, along with other reagents required for phytochemical analysis, were utilized in the study. Diclofenac and Glibenclamide, used as reference drugs for the analgesic and hypoglycemic assays, were procured from a local pharmaceutical supplier in Bangladesh.

### 2.5 Experimental Animal

Swiss albino mice, approximately 4–5 weeks old and weighing between 25 and 30 g, were used in the study. The mice were sourced from Jahangirnagar University, Bangladesh, and were housed under controlled conditions, maintaining a temperature of 25°C ± 2°C and relative humidity of 60%–70% for a period of 14 days. During this acclimatization period, they were provided with a standard diet and ad libitum access to clean water. All experimental procedures were approved by the Ethics Approval Committee for Animal Handling at Manarat International University, under approval number (MIU/SEST/ERC/2024002).

### 2.6 Statistical Analysis

A version 12 of the Statistical Package for the Social Sciences (SPSS) for Windows was used to conduct the statistical analysis. The data was shown as the average plus or minus the standard deviation or as a percentage where relevant. To find out whether the differences in values were statistically significant, the unpaired *t* test was used. Additionally, the measurements showed a connection. Statistical significance was determined by a two‐tailed *p*‐value less than 0.05.

### 2.7 Evaluation of Hypoglycemic Activity

A freshly prepared solution of streptozotocin (STZ) at a dose of 50 mg/kg body weight, dissolved in citrate buffer (0.1 M, pH 4.5), was administered intraperitoneally to induce diabetes in the mice. Following the STZ injection, the animals were given a 10% glucose solution for 2 h to prevent hypoglycemic shock. Hyperglycemic assessments were conducted 1 week after STZ administration. Mice exhibiting fasting blood glucose levels exceeding 230 mg/dL were selected for the hypoglycemic studies. Blood glucose levels were monitored using an Accu‐Chek meter (Roche).

#### 2.7.1 Glucose Tolerance Test

A total of 20 mice were chosen and divided into four groups consisting of five mice in each group. To prepare the standard (Glibenclamide) at a dosage of 10 mg/kg body weight, a 10 mg tablet was dissolved in 3.0 mL of normal saline (0.9% NaCl).

Group 1—Normal Control.

Group 2—Diabetic Control.

Group 3—Diabetic + Glibenclamide (50 mg/kg b.w.).

Group 4—Diabetic + Test sample (200 mg/kg b.w.).

After 1 h of administering the drug, mice in the normal and diabetic groups were given 10 mg/kg of glucose. Blood samples were obtained from all mice's tail veins at 0, 30, 60, 90, and 120 min. A glucometer was used to estimate the blood glucose level.

#### 2.7.2 Acetic Acid‐Induced Writhing Test

The evaluation was conducted using the technique outlined by Koster et al. Four groups, each consisting of five mice, were used to evaluate analgesic activity by the writhing test. Group 1 (positive control) was administered 100 mg/kg of diclofenac sodium orally. Group 2 received distilled water orally as the negative control. Groups 3 and 4 were administered oral medications at dosages of 200 and 400 mg/kg, respectively. Each animal received an intraperitoneal injection of 0.6% (v/v) acetic acid at a dosage of 10 mL/kg body weight. Various writhing responses from each animal were recorded throughout a 5‐min interval and 15 min after the injection of acetic acid. The mean level of abdominal writhing for each group was also determined. The inhibition % of the writhing response was calculated as follows: Percentage (%) is calculated as 100 multiplied by the difference between the mean frequency of contractions in the negative control animals (Cn) and the mean frequency of contractions in the animals administered diclofenac sodium and different MEDS concentrations (Ct), divided by Cn.

### 2.8 In Silico Study

#### 2.8.1 Protein Preparation, Molecular Docking, Analysis and Visualization

The 3D crystal structures of human cyclooxygenase‐2 (PDB ID: 5KIR) (21) and sulfonylurea receptor‐1 (PDB ID: 6JB3) (Medha et al. 2022) were collected in PDB format from the protein databank (PDB). The software package Discovery Studio Visualizer (V16.1.0.15350) was used to remove heteroatoms and water molecules from the protein chain, which were then remounted for energy minimization by Swiss PdbViewer (Version 4.1.0) software. The optimized structures were then molecularly docked using the PyRx (Version 0.8) software tool on both PDB ID: 5KIR and PDB ID: 6JB3, with the protein serving as the macromolecule and medicines as the ligands. The COX‐2 protein has center grid box sizes of 78.7416 Å, 62.0367 Å, and 64.0263 Å in the *x*‐, *y*‐, and *z*‐axes, respectively. The grid box enclosed the entire protein. The SUR‐1 protein had a center grid box size of 77.0448 Å, 61.0577 Å, and 87.5629 Å in the *x*, *y*, and *z* directions, respectively. Discovery Studio (Version 4.1) was used to design nonbonded interactions, as well as to evaluate and visualize docking consequences (Alesawy et al. 2021). LigPlot+ has been used to visualize and analyze protein‐ligand interactions, which generate 2D schematic diagrams of ligand‐protein complexes, highlighting key interactions such as hydrogen bonds and hydrophobic contacts.

## 3 Results and Discussion

### 3.1 Glucose Tolerance Test

**FIGURE 1:**
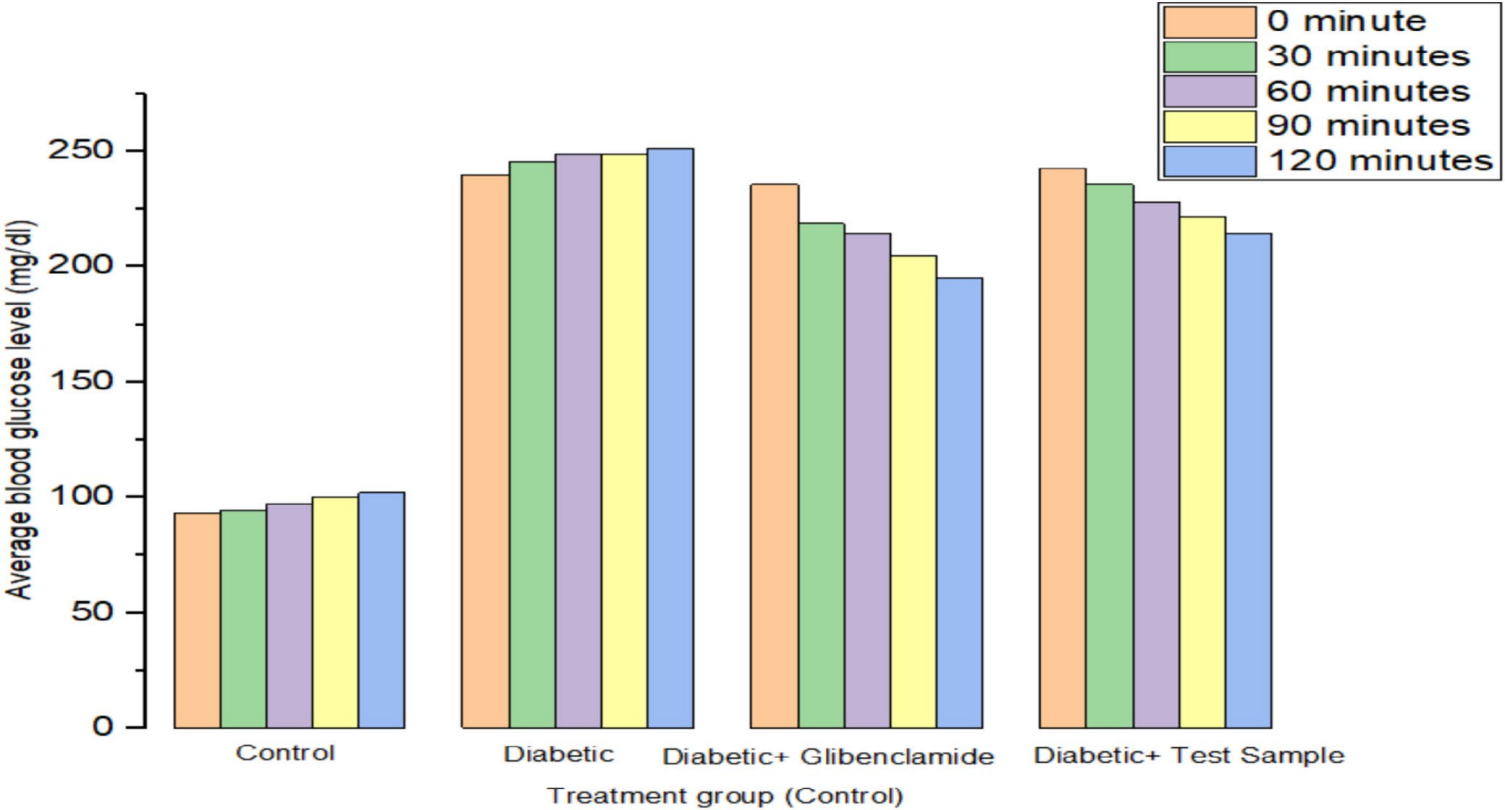
Serum glucose level by glucose tolerance test.

Effect of the extract of MECG on blood glucose levels of diabetic and normal mice over 2 h have been shown in Table 1. The study's findings indicated that the methanolic extract exhibited significant hypoglycemic activity in both STZ‐induced diabetic mice and normoglycemic mice. Serum glucose levels decreased by 18.66% with Glibenclamide and by 10.57% with the methanolic extract at doses of 200 mg/kg, compared to the diabetic control group, as shown in Table 1 and Figure 1.

**TABLE 1:** Effect of MECG on serum glucose level.

Groups	Average blood glucose level (mg/dL)				
	0 min	30 min	60 min	90 min	120 min
Normal control	93.39 ± 1.84	94.66 ± 1.22	97.11 ± 1.07	99.84 ± 0.78	102.11 ± 0.77
Diabetic control	239.86 ± 0.45	245.46 ± 0.65	248.71 ± 0.30	249.04 ± 0.24	251.11 ± 0.37
Diabetic + Glibenclamide (50 mg/kg b.w.)	235.46 ± 0.46	218.66 ± 0.48	214.316 ± 0.48	204.44 ± 0.48	195.11 ± 0.41
Diabetic + test sample (200 mg/kg b.w.)	242.46 ± 0.42	235.46 ± 0.38	227.91 ± 0.63	221.84 ± 0.54	214.51 ± 0.44

### 3.2 Evaluation of Analgesic Activity

#### 3.2.1 Acetic Acid‐Induced Writhing Method

**FIGURE 2:**
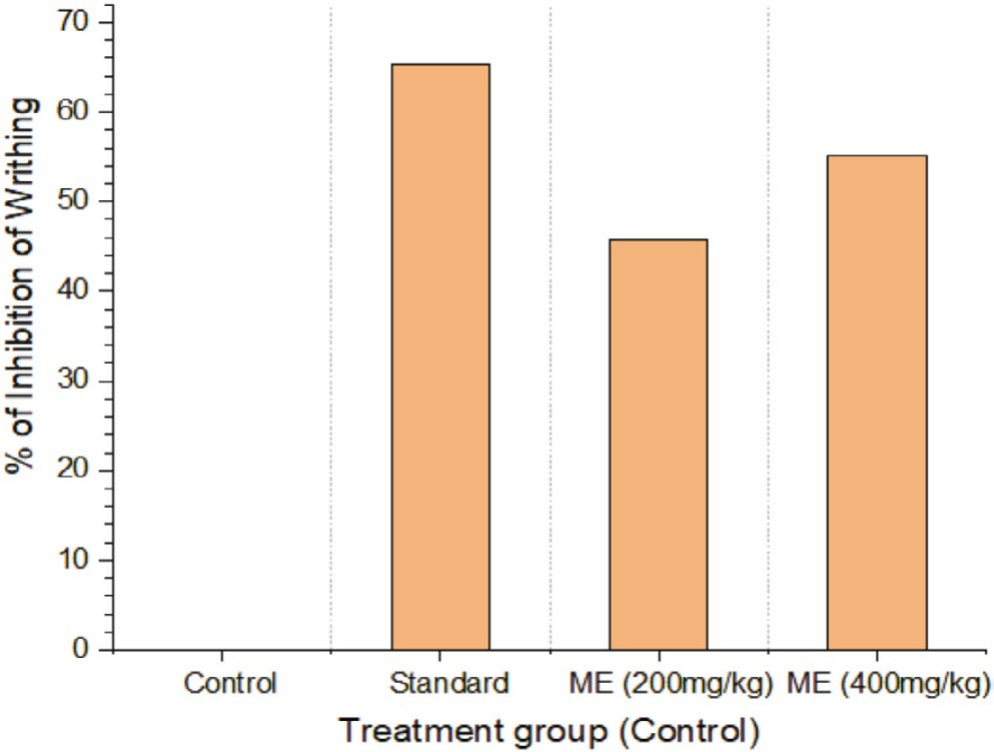
Effect of MECG using the writhing method.

The treated rodents did not writhe significantly more than the control group in the acetic acid‐induced writhing assay. Nevertheless, the levels of writhing induced by diclofenac sodium were nearly identical to those of the extract‐treated groups. The extract has a dose‐dependent antinociceptive effect on the writhing of rodents induced by acetic acid, as demonstrated in Table 2 and Figure 2.

**TABLE 2:** Effects of MECG in acetic acid‐induced writhing test.

Treatment group	Number of Writhing (Mean ± SEM)	*n*	% of Inhibition of Writhing
Control	25.4 ± 0.6	5	0
Standard	8.8 ± 0.6633	5	65.35
ME (200 mg/kg)	13.8 ± 0.7348	5	45.66
ME (400 mg/kg)	11.4 ± 0.4	5	55.11

*Note:* Data are given as the mean ± SEM (*n* = 5). ****p < 0.0001.

Abbreviation: ME, Methanolic extract.

### 3.3 In Silico ADME Analysis

Table 3 displays the physicochemical parameters needed for MECG isolated chemicals were studied using ADME. The compounds are identified as potential drug candidates because they all adhere to the Lipinski rule (Chen et al. 2020) which requires a molecular weight of less than 500 g/mol, fewer than ten H‐bond acceptors, and fewer than five H‐bond donors. Here all other compounds matched the criteria to be classified as drug molecules. Diclofenac sodium, and Glibenclamide (a co‐crystallized ligand of 6JB3) (as a standard used in vivo investigations) were all orally delivered medications that did not break Lipinski's criterion (RO5).

**TABLE 3:** ADME and toxicity analysis of the phytoconstituents isolated from MECG.

Chemical name	MW (g/mol)	HBA	HBD	i log *p*	No. of violations
d‐mannitol, 1‐decyl sulfonyl	387.51	8	7	1.42	1
Alpha‐monopropionin	148.16	4	2	1.62	0
3‐Ethoxy‐ 1,2‐propanediol	120.15	3	2	1.69	0
2‐Cyclo penten‐1‐one	84.12	1	0	1.38	0
3,3‐Dimethoxy‐2‐butanone	132.16	3	0	1.58	0
3‐Methylbenzyl alcohol	122.16	1	1	1.94	0
Benzoic acid,2‐methoxy‐, methyl ester	166.17	3	0	2.13	0
Benzamide, N‐(2‐methoxyacetyl)	193.20	3	1	1.29	0
2,2′‐oxybis(ethan‐1‐ol)	106.12	3	2	1.48	0
3‐Ethoxypropanol	104.15	2	1	1.74	0
1‐Hexanol, 2‐ethyl—	130.23	1	1	2.50	0
Nonadecane	268.52	0	0	5.46	1
2‐Methoxy‐4‐vinyl phenol	150.17	2	1	2.14	0
3‐hydroxy phenylacetic acid	152.15	3	2	1.02	0
Cyclopropane butanoic acid, 2‐[[2‐[[2‐[(2pentylcyclopropyl) methyl] cyclopropyl] methyl] cyclopropyl] methyl]‐, methyl ester	374.60	2	0	5.20	1
Methyl tetra decanoate	242.40	2	0	3.88	0
Dodecane, 2,6, 11‐trimethyl—	212.41	0	0	4.15	1
Z‐2‐Octadecen‐1‐ol acetate	310.51	2	0	5.32	1
Hexadecenoic acid, methyl ester	270.45	2	0	4.41	1
Benzene propanoic acid, 3,5‐bis(1,1‐dimethylethyl)‐4‐hydroxy‐, methyl ester	292.42	3	1	4.09	0
Methyl octyl phthalate	292.37	4	0	3.34	0
Methyl10‐trans,12‐cis octadecadienoate	298.50	2	0	4.81	1
6‐octadecenoic acid, methyl ester, (Z)—	296.49	2	0	4.70	1
11‐Octadecenoic acid, methyl ester	296.49	2	0	4.79	1
Phytol	296.53	1	1	4.84	1
Methyl stearate	298.50	2	0	4.81	1
Lanosterol	426.72	1	1	5.09	1
cis‐9‐Hexadecenal	238.41	1	0	3.98	1
cis‐10‐Nonadecenoic acid, methyl ester	310.51	2	0	5.27	1
Bis(2‐ethylhexyl) phthalate	390.56	4	0	4.77	1
Tetracosanoic acid, methyl ester	382.66	2	0	6.32	1
13‐Docosenamide, (Z)—	337.58	1	1	5.02	1
Squalene	410.72	0	0		
tert‐butyl (2‐aminophenyl) carbamate	208.26	2	2	2.19	0
2‐(methoxymethyl)‐4‐oxopentanoic acid	160.17	4	1	1.1	0
Pyrazine, 2,5‐bis(1,1‐dimethylethyl)‐, 1,4 dioxide	224.30	2	0	1.84	0
3,4‐Dihydro‐3,5,8‐trimethyl‐3‐(4,8,12‐trimethyltridecyl)‐(2H)1‐benzopyran‐6‐acetate	458.72	3	1	5.43	1
2,6‐Lutidine3,5‐dichloro‐4dodecylthio—	376.43	1	0	5.15	1
alpha‐Tocopherol acetate	472.74	3	0	6.34	1
Cholesterol	386.65	1	1	4.96	1
Stigmasterol	412.69	1	1	5.01	1
beta‐Sitosterol	414.71	1	1	4.79	1

### 3.4 In Silico Study for the Assessment of Analgesic Activity

**FIGURE 3:**
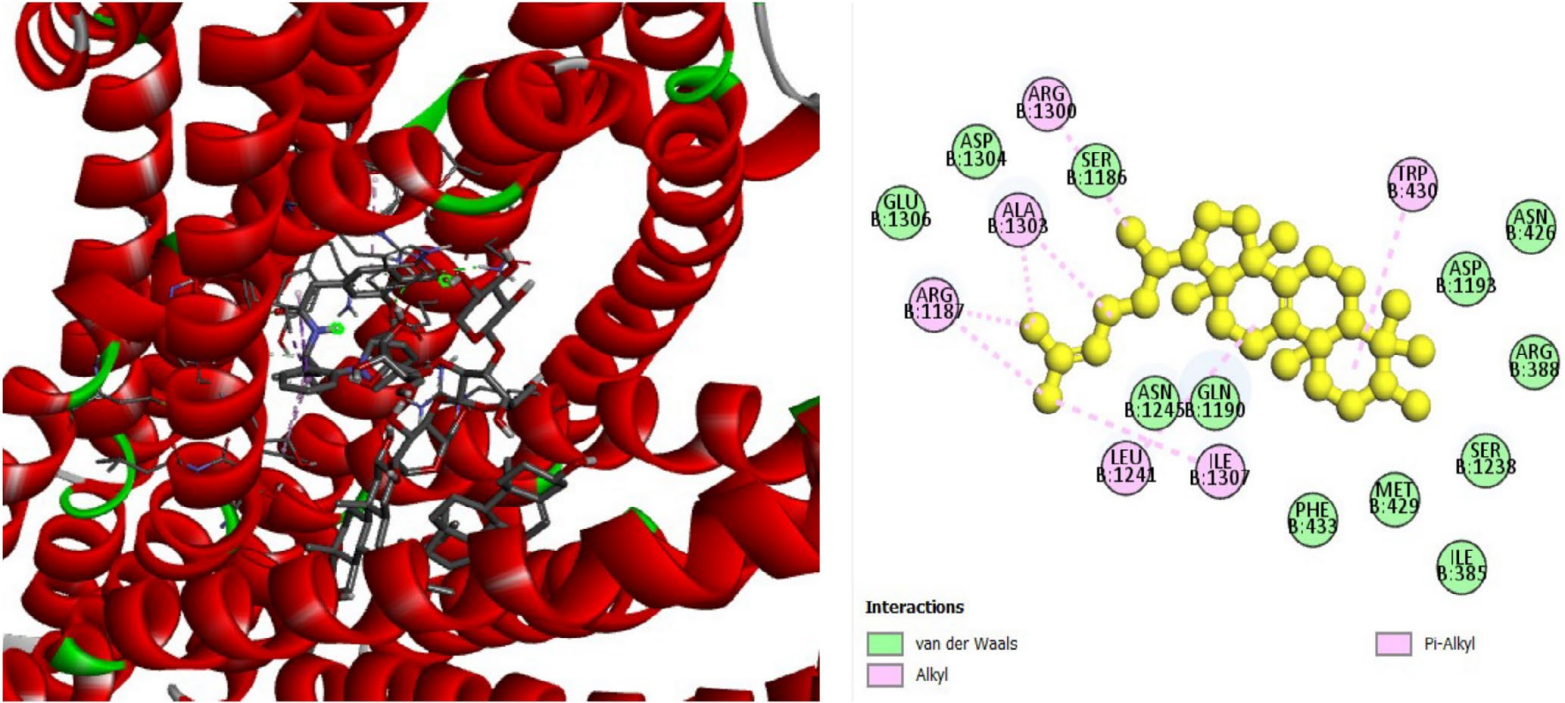
Lanosterol with the inhibitory site of the 5KIR protein.

**FIGURE 4:**
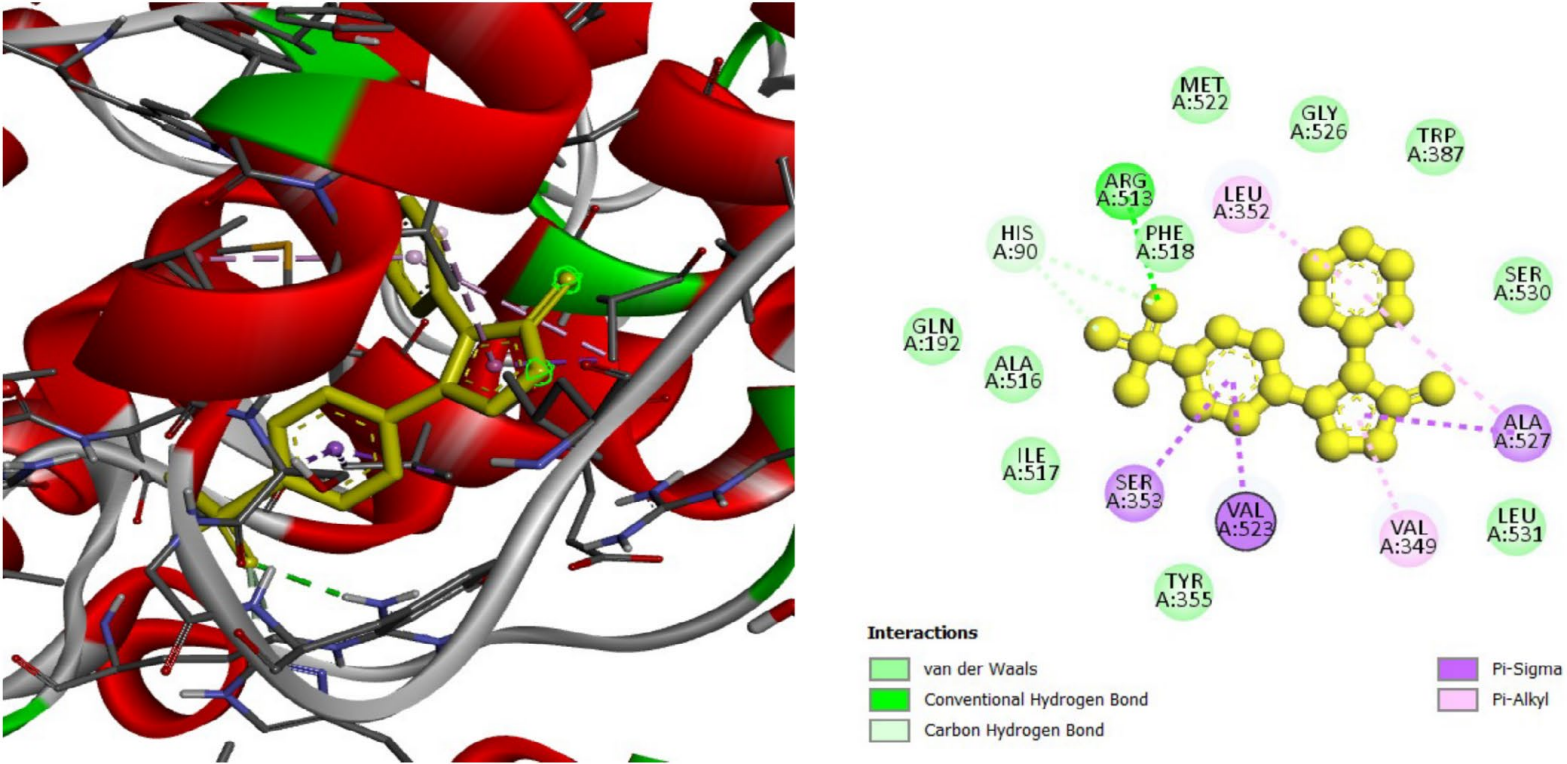
Stigmasterol with the inhibitory site of the 5KIR protein.

**FIGURE 5:**
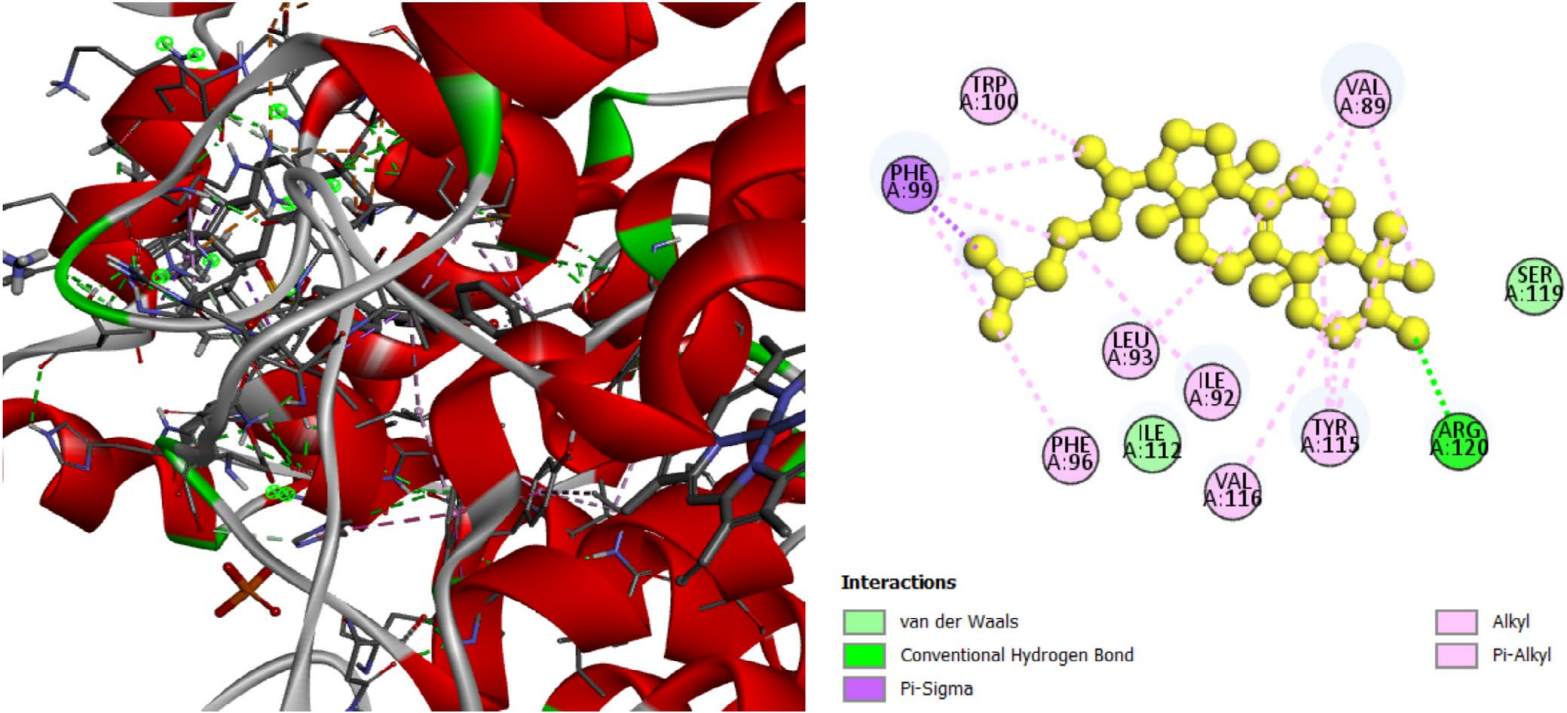
Beta‐Sitosterol with the inhibitory site of 5KIR protein.

The analgesic potential of COX2 (PDB ID: 5KIR) receptors was studied using molecular docking simulations. Table 4 summarizes the docking scores. Diclofenac sodium, the reference drug, had lower docking scores of −6.7 kcal/mol, Table 4 shows the interactions of the phytochemicals and the standard drugs diclofenac sodium with the COX‐2 protein, as well as their binding interactions with the individual amino acids of the protein's inhibitory region. The docking value for stigmasterol is −7.9 kcal/mol, making it the lowest in the table. Figures 3, 4, 5 shows the 2D binding of Lanosterol, Stigmasterol, and beta‐Sitosterol with inhibitory site of 5KIR receptor.

**TABLE 4:** Molecular docking results against COX‐2 (5KIR).

Compound	Docking Score (kcal/mol)	Interactions	
		Hydrogen bonds in Å	Hydrophobic bonds in Å
Diclofenac	−6.7	ARG120 (2.3)	VAL89 (3.6), TYR115 (5.0), ILE112 (4.8)
d‐mannitol, 1‐decyl sulfonyl	−6.7	ASN43 (2.2), ARG44 (2.0), CYS47 (2.6), GLN461 (2.3), GLU465 (2.4), GLU465 (2.9), CYS41 (2.0)	
Alpha‐monopropionin	−4.7	THR206 (1.9), HIS386 (3.7)	HIS386 (3.9)
3‐Ethoxy‐ 1,2‐propanediol	−4.3	TYR385 (2.4), HIS207 (3.4)	
2‐Cyclo penten‐1‐one	−4.5		GLN203 (4.0), ALA202 (4.0)
3,3‐Dimethoxy‐2‐butanone	−4.7	ALA52 (72.7), SER53 (1.9)	
3‐Methylbenzyl alcohol	−5.9	THR206 (2.5), TYR385 (3.5)	GLN20 (4.8), ALA199 (3.6), LEU390 (4.2), LEU391 (4.4) ALA202 (5.0)
Benzoicacid,2‐methoxy‐, methyl ester	−6	TYR355 (2.3), SER353 (3.7), SER353 (3.4)	VAL523 (3.9), LEU352 (4.9), ALA527 (5.0)
Benzamide, N‐(2‐methoxyacetyl)	−7.5		ALA202 (5.1)
2,2′‐oxybis(ethan‐1‐ol)	−4.1	ALA199 (2.6), TYR385 (2.2)	
3‐Ethoxypropanol	−4.1	GLN203 (3.0), TYR385 (2.5)	
1‐Hexanol, 2‐ethyl—	−4.4		LYS468 (5.3), ARG469 (4.6)
Nonadecane	−4.8		PRO156 (3. 9), HIS39 (5. 0)
2‐Methoxy −4‐vinyl phenol	−6.3	SER530 (2.4), SER530 (1.8), TYR385 (3.5)	LEU352 (4.4), VAL523 (4.0), PHE518 (4.9), LEU3 (4.9), VAL523 (5.2)
3‐hydroxy phenylacetic acid	−6.1	SER530 (2.2), VAL523 (2.4), LEU352 (2.6)	VAL523 (3.7), LEU352 (5.0), ALA527 (5.3)
Cyclopropane butanoic acid, 2‐[[2‐[[2‐[(2pentylcyclopropyl) methyl] cyclopropyl] methyl] cyclopropyl] methyl]‐, methyl ester	−6.9	ARG120 (3.0), ARG120 (2.9), GLU524 (3.6)	PRO84 (5.1), VAL89 (4.3), VAL89 (4.6), ILE92 (3.9), LEU93 (4.6) ILE112 (4.9), ILE112 (4.1), PHE99 (5.2), TRP100 (5.4), TRP100 (4.5), TYR115 (4.9)
Methyl tetra decanoate	−5.1	ARG469 (3.6), AG469 (3.5)	CYS36 (4.1), CYS47 (4.0), HIS39 (5.0)
Dodecane, 2,6, 11‐trimethyl—	−5.2		VAL116 (3.8), ARG120 (5.0) PRO86 (3.9), VAL89 (4.1), PRO86 (4.5), VAL89 (3.7) ILE112 (3.9), VAL89 (5.1), LEU93 (4.1) TRP100 (5.4), TRP100 (5.1)
Z‐2‐Octadecen‐1‐ol acetate	−5.6	TYR130 (2.1), VAL46 (3.5)	PRO154 (4.4)
Hexadecenoic acid, methyl ester	−5	ARG44 (2.5)	LEU152 (4.8), PRO153 (4.6), TYR130 (5.0)
Benzene propanoic acid, 3,5‐bis(1,1‐dimethylethyl)‐4‐hydroxy‐, methyl ester	−6.6		CYS36 (4.8), PRO153 (4.5), CYS36 (5.4), PRO156 (4.4)
Methyl octyl phthalate	−5.8	SER119 (2.6), ARG120 (2.8), ARG120 (2.6)	VAL89 (3.9), ILE92 (3.9), PRO86 (4.9), VAL89 (4.1)
Methyl10‐trans,12‐cis octadecadienoate	−5.6	ARG44 (1.9), ARG469 (3.6), ALA151 (3.6)	
6‐octadecenoic acid, methyl ester, (Z)—	−5.5	ARG44 (2.1), ARG44 (2.0), ARG469 (2.3), ASP125 (3.5)	PRO156 (4.6)
11‐Octadecenoic acid, methyl ester	−5.4	HIS388 (3.6), HIS388 (3.2)	VAL291 (3.6), LEU294 (5.2), HIS207 (5.0)
Phytol	−5.6		VAL89 (4.9), LEU93 (5.0) VAL116 (4.4), PRO86 (4.6), VAL89 (3.7) ILE112 (3.9), VAL89 (3.7) ILE92 (4.7), VAL89 (4.1), ILE92 (4.0), LEU9 (3.9), TYR115 (4.9)
Methyl stearate	−5	LYS83 (2.2)	
Lanosterol	−7.8		LEU145 (5.1), LEU145 (4.7), VAL538 (4.3), LEU145 (4.2), TRP139 (5.0), TRP139 (5.1), PHE (4.9), HIS226 (4.6)
cis‐9‐Hexadecenal	−5	ASN43 (2.6), ARG44 (2.2), PRO153 (4.4)	HIS39 (4.2)
cis‐10‐Nonadecenoic acid, methyl ester	−4.9		PRO84 (4.9), VAL89 (3.6), ILE92 (4.2)
Bis(2‐ethylhexyl) phthalate 35	−5.9	HIS207 (2.4), THR212 (1.9), GLN289 (2.2)	HIS207 (5.3), LYS211 (4.0), VAL447 (4.3), HIS207 (4.6), PHE210 (5.0), HIS214 (5.2), HIS386 (5.1), HIS386 (4.9), VAL291 (4.9)
Tetracosanoic acid, methyl ester	−6.3	SER530 (2.1)	VAL89 (3.6)
13‐Docosenamide, (Z)—	−5	ARG120 (2.9), ARG120 (2.9), GLU524 (3.0)	ILE92 (4.2)
Squalene	−6.8		ILE92 (4.9), LYS83 (4.1), LYS83 (4.1), PRO84 (4.98), VAL89 (5.2), LEU93 (4.6), VAL116 (4.2), PRO86 (4.8), VAL89 (3.7), LEU123 (4.4), MET471 (4.5), TRP100 (4.9)
tert‐butyl (2‐aminophenyl) carbamate	−5.7	LYS468 (2.6), ASP499 (2.6), ASP499 (2.2)	HIS39 (3.8), LYS166 (3.9)
2‐(methoxymethyl)‐4‐oxopentanoic acid	−5	ARG44 (3.4)	
Pyrazine, 2,5‐bis(1,1‐dimethylethyl)‐, 1,4‐dioxide	−6.4		CYS36 (4.5), PRO153 (4.9), PRO156 (4.9), CYS47 (4.9), PRO153 (4.0)
3,4‐Dihydro‐3,5,8‐trimethyl‐3‐(4,8,12‐trimethyltridecyl)‐(2H)1‐benzopyran‐6‐acetate	−7.3		VAL89 (3.5), PHE96 (3.8), TYR115 (5.0), VAL89 (4.8), LEU93 (5.3), VAL89 (4.9), LEU93 (5.4), VAL116 (4.5), LEU93 (4.6) ILE112 (4.1), PRO84 (4.1), ILE92 (4.5), ILE92 (3.7), PHE96 (4.5), TRP100 (5.0), TRP100 (4.9) TYR115 (5.0)
2,6‐Lutidine3,5‐dichloro‐4dodecylthio—	−5.5		VAL89 (4.2), VAL89 (4.4), LYS83 (4.6) PRO84 (4.9), ILE112 (4.0), TYR115 (5.3), TYR115 (4.5), VAL89 (4.3)
alpha‐Tocopherol acetate	−7.3	SER119 (2.6)	VAL89 (13.6), PHE99 (3.9), PHE96 (3.7), TYR115 (5.0), VAL89 (5.3), VAL116 (5.3), LEU93 (4.57), ILE112 (4.3), VAL89 (4.9), PRO84 (4.3), VAL89 (3.6), ILE92 (4.4), ILE92 (4.5), PHE96 (4.12), PHE99 (5.0), TRP100 (5.3), TRP100 (4.5), TYR115 (5.2), TYR115 (5.4)
Cholesterol	−7.5		ALA111 (3.7), ILE112 (4.2), ILE112 (3.9), ILE11 (5.1), VAL89 (5.2) LEU93 (4.1), PRO84 (4.1), VAL89 (4.8), ILE92 (4.6), PRO84 (4.7), VAL89 (3.8), TYR115 (5.2)
Stigmasterol	−7.9		VAL447 (5.3), LEU294 (4.4), VAL447 (4.5), VAL447 (5.0), LEU294 (4.5), VAL295 (4.7), LEU391 (5.2), ILE408 (5.1), ILE408 (4.2), HIS207 (4.5), HIS207 (4.3), HIS386 (5.1), HIS388 (5.2), TYR404 (4.9), TYR404 (5.2)
beta‐Sitosterol	−7.2		ALA111 (3.7), ILE112 (3.9), ILE112 (4.0), ILE112 (5.2), LEU93 (4.3), ILE112 (5.3), PRO84 (4.6), VAL89 (3.9), PRO84 (3.9), VAL89 (4.3), ILE92 (4.6), TYR115 (5.2)

### 3.5 In Silico Study for the Assessment of Antidiabetic Activity

**FIGURE 6:**
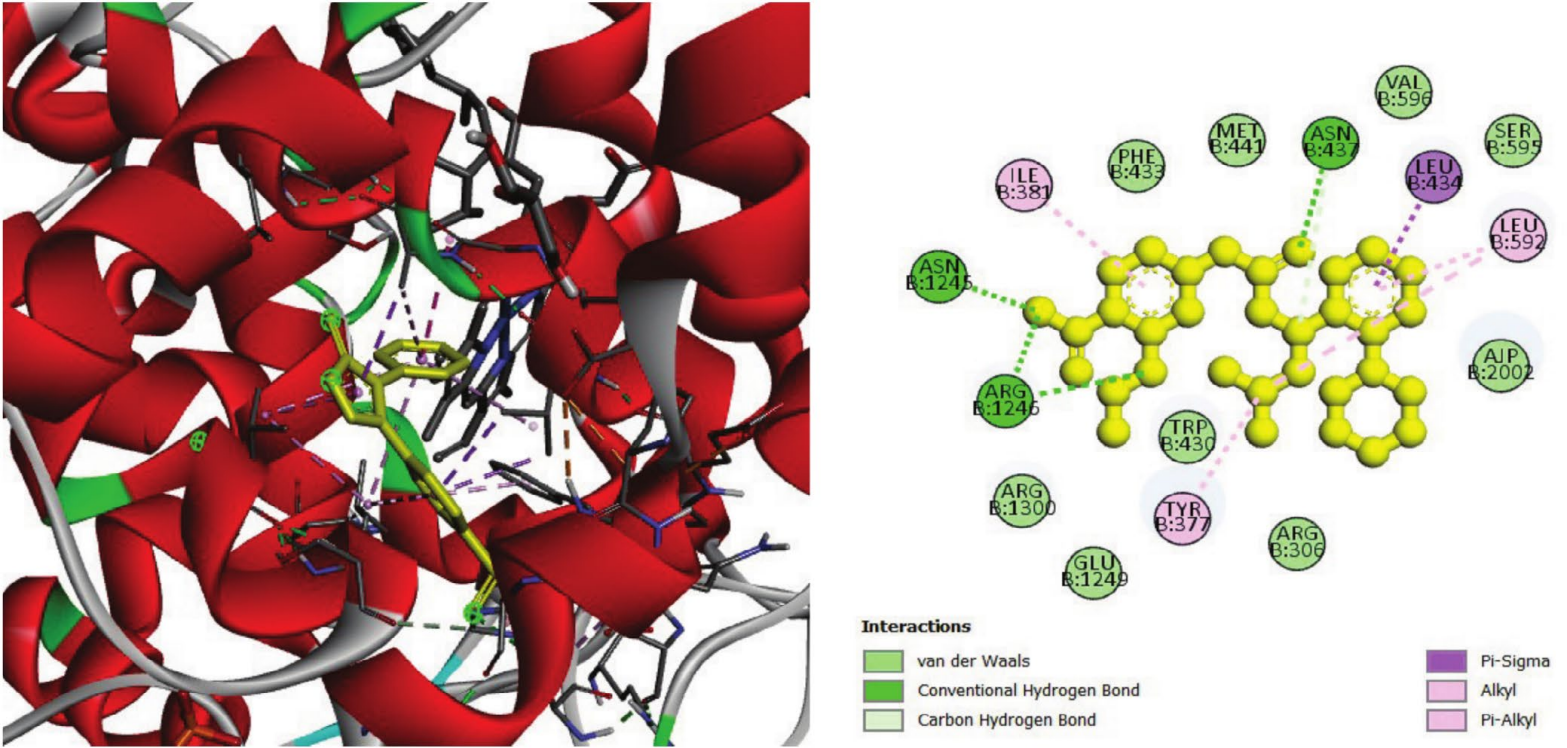
Lanosterol with the inhibitory site of the 6JB3 protein.

**FIGURE 7:**
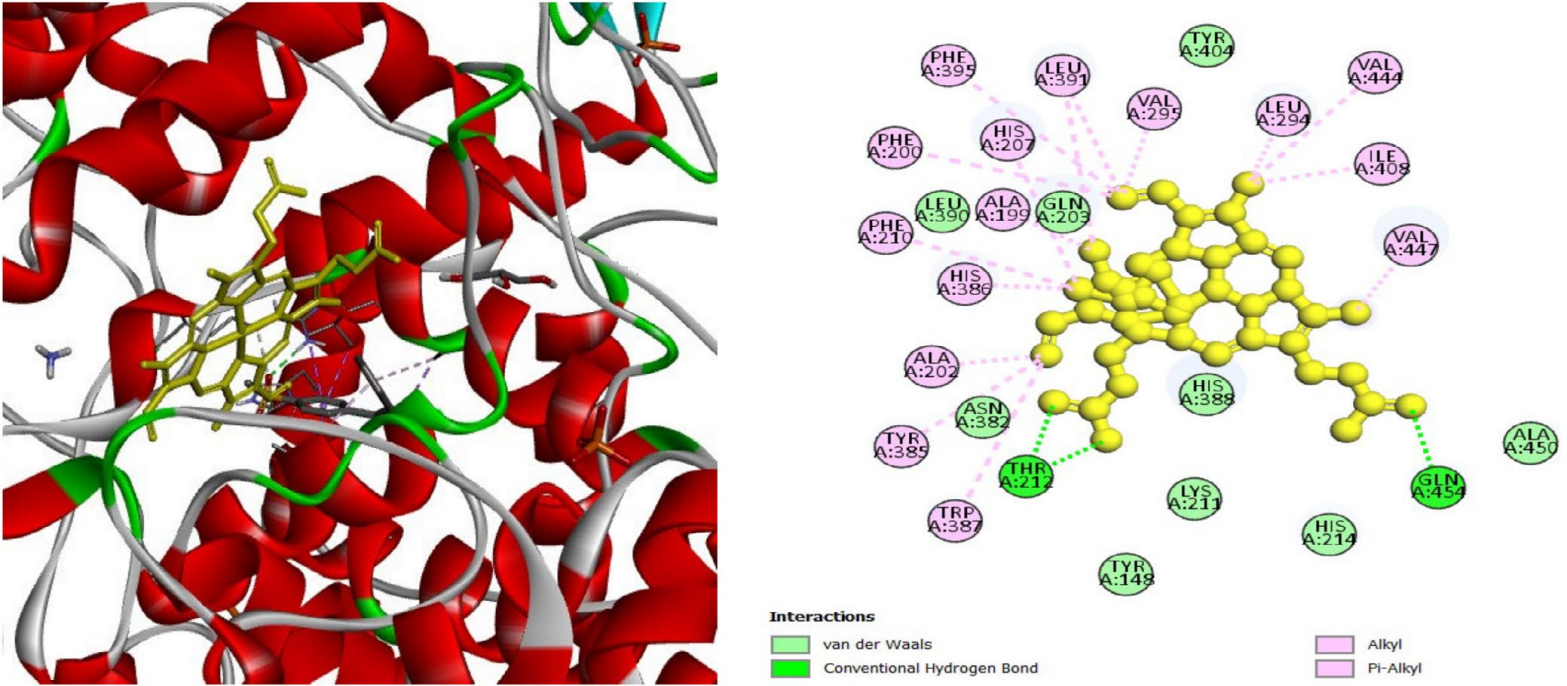
Stigmasterol with the inhibitory site of the 6JB3 protein.

**FIGURE 8:**
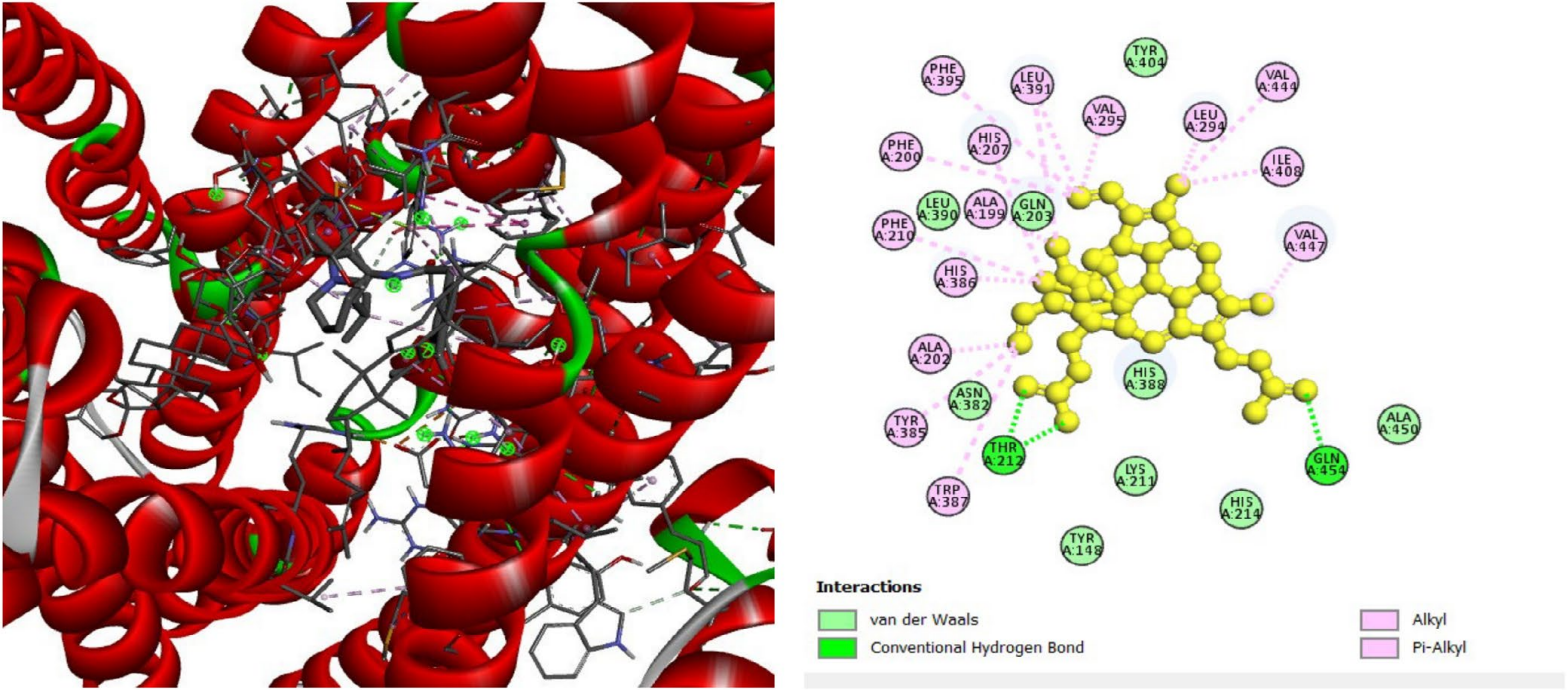
Beta‐Sitosterol with the inhibitory site of the 6JB3 protein.

To investigate the hypoglycemic potential, molecular docking simulations were performed on the SUR1 (PDB ID: 6JB3) receptor. The best docking results are shown in Table 5. Lanosterol has the lowest docking score (−10.3 kcal/mol), as shown in Table 5. The standard drug, Glibenclamide, had docking scores of −9.4. Table 5 describes the interactions of phytochemicals and the standard drug Glibenclamide with the SUR1 receptor, as well as their binding interactions with specific amino acids in the protein's inhibitory region. Figures 6, 7, 8 show the 2D binding of Lanosterol, Stigmasterol, and beta‐Sitosterol with the inhibitory site of the 6JB3 receptor.

**TABLE 5:** Molecular docking results against SUR‐1 (6JB3).

Compound	Docking Score (kcal/mol)	Interactions	
		Hydrogen bonds in Å	Hydrophobic bonds in Å
Glibenclamide	−9.4	ASN547 (2.3), ARG1300 (2.6), ARG1300 (2.7)	VAL587 (3.7), TRP1297 (4.1), TRP1297 (3.9), PRO551 (3.7), PRO551 (5.3)
d‐mannitol, 1‐decyl sulfonyl	−5.7	GLN374 (2.7), THR588 (2.1), ASN1296 (2.9), ARG1300 (2.9), GLU1253 (2.3), GLU1249 (2.0), ASN1296 (3.5)	TYR1294 (4.8), TRP1297 (4.5)
Alpha‐monopropionin	−4.5	ASN1245 (2.8), ARG1300 (2.4)	
3‐Ethoxy‐ 1,2‐propanediol	−4	GLN369 (2.3), GLN369 (1.9), GLU1253 (2.1)	
2‐Cyclo penten‐1‐one	−4		ARG1187 (4.8), ALA1303 (4.3), ILE1307 (4.8)
3,3‐Dimethoxy‐2‐butanone	−4	GLN444 (2.5), GLN4442 (3.0), ALA309 (3.5)	
3‐Methylbenzyl alcohol	−5	ASN234 (2.3)	TYR1181 (4.2), PHE1177 (4.8), TYR1181 (4.6)
Benzoic acid,2‐methoxy‐, methyl ester	−5.5	THR413N (1.7)	PHE613 (3.8), LEU420 (4.7), LYS609 (5.3)
Benzamide, N‐(2‐methoxyacetyl)	−6.6	ASN547 (2.1), ASN5471 (2.8), THR548 (2.2), ARG1145 (2.4)	TRP1297 (4.7), TRP129 (3.7)
2,2′‐oxybis(ethan‐1‐ol)	−3.5	LEU503 (2.6)	
3‐Ethoxypropanol	−3.6	ARG370 (2.3), GLU1253 (2.6)	
1‐Hexanol, 2‐ethyl—	−4.3		ARG1187 (3.9), ILE1307 (3.8)
Nonadecane	−5.1		VAL587 (5.2), PRO551 (5.3), ILE552 (4.5), VAL555 (4.6), HIS584 (5.0)
2‐Methoxy −4‐vinyl phenol	−5.3	ARG370 (2.3), ARG370 (2.2), GLU1253 (2.3), GLN369 (2.6)	ILE585 (3.9)
3‐hydroxy phenylacetic acid	−5.5	LYS609 (2.5)	PHE613 (3.5) PHE613 (5.0), LEU420 (4.8), LYS609 (5.3)
Cyclopropane butanoic acid, 2‐[[2‐[[2‐[(2pentylcyclopropyl) methyl] cyclopropyl] methyl] cyclopropyl] methyl]‐, methyl ester	−6.4	ASN547 (2.7), ASN547 (3.5) THR548 (3.6)	LEU592 (4.7), HE591 (4.4), TRP1297 (4.4), TRP1297 (4.1), TRP1297 (5.3), TRP1297 (4.6), TRP1297 (4.7)
Methyl tetra decanoate	−5	ASN547 (2.3), ARG1145 (2.9)	TRP1297 (3.7), PHE59 (3.9), VAL587 (5.1), PHE591 (4.5)
Dodecane, 2,6, 11‐trimethyl—	−4.9		HIS584 (3.7), MET129 (5.2), LEU1027 (4.2), LEU1027 (4.8), LEU1149 (4.8) HIS584 (5.1), TRP1297 (4.9)
Z‐2‐Octadecen‐ 1‐ol acetate	−5.6	ASN547 (2.6), THR548 (2.2), ARG1145 (2.4)	LEU592 (4.1), PHE591 (4.6)
Hexadecenoic acid, methyl ester	−5	ASN547 (2.3)	ILE552 (4.3), VAL555 (4.4) HIS584 (5.4)
Benzenepropanoic acid, 3,5‐bis(1,1‐dimethylethyl)‐4‐hydroxy‐methyl ester	−7.2	ASN547 (1.9), THR588 (2.3), HIS584 (3.5)	TRP1297 (4.2), LEU1149 (5.4), TRP1297 (4.3)
Methyl octyl phthalate	−5.9		TRP1297 (3.65), PHE591 (4.8), TRP1297 (4.4), LEU592 (4.7)
Methyl10‐trans,12‐cis octadecadienoate	−5.7	ASN547 (2.7), THR548 (2.3), ARG1145 (2.4)	TRP1297 (4.6)
6‐octadecenoic acid, methyl ester, (Z)—	−5.2	ASN547 (2.4), ARG1145 (2.7), ARG1145 (3.5)	TRP1297 (4.3)
11‐Octadecenoic acid, methyl ester	−5.1		TRP1297 (3.7), TRP1297 (4.1)
Phytol	−5.6	THR588 (2.5)	TRP1297 (3.5), LEU59 (4.6), VAL587 (4.6), LEU1149 (5.2), VAL587 (4.54), PHE591 (4.8), TYR1294 (4.9), TYR1294 (4.9), TRP1297 (4.9), TRP1297 (4.2)
Methyl stearate	−4.7	ASN547 (3.3)	
Lanosterol	−10.3		TRP1297 (3.8), LEU1149 (5.4), VAL587 (4.3), LEU1149 (4.6), LEU592 (4.3), LEU1027 (4.9), LEU1149 (5.1), ILE552 (4.3), VAL555 (4.2), LEU1027 (4.0), HIS584 (5.0), PHE591 (4.1), PHE591 (5.1), TYR1294 (5.4), TYR1294 (4.4), TRP1297 (5.1), TRP1297 (5.1), TRP1297 (4.4), TRP1297 (4.4), TRP1297 (4.) TRP1297 (4.3), TRP1297 (4.7)
cis‐9‐Hexadecenal	−5.1	ASN547 (2.6), THR548 (2.3), ARG1145 (2.5)	TRP1297 (3.7), PHE591 (4.5), TRP1297 (4.9)
cis‐10‐Nonadecenoic acid, methyl ester	−4.7		TYR1294 (4.0), TRP1297 (4.5)
Bis(2‐ethylhexyl) phthalate	−6.7	ASN547 (2.2), ASN547 (2.4), ARG1145 (2.8), ASN547 (3.5)	TRP1297 (3.5), PRO551 (4.3), ILE552 (4.6), VAL587 (3.7), CYS1142 (4.9), HIS584 (4.8), PHE591 (4.6), TRP1297 (4.2), TRP1297 (4.5), TRP1297 (5.3)
Tetracosanoic acid, methyl ester	−5.1	THR588 (2.5), ASN1293 (2.), HIS584 (3.4)	
13‐Docosenamide, (Z)—	−5.9	THR588 (2.7), ASN1293 (2.6)	ILE552 (4.2), VAL555 (3.9) ILE1030 (5.4)
Squalene	−7.3		LEU592 (5.1), LEU592 (4.0), VAL587 (4.7), VAL587 (4.0), VAL587 (5.1), HIS584 (4.3), PHE591 (4.7), PHE591 (4.8), TRP1297 (4.9)
tert‐butyl (2‐aminophenyl) carbamate	−5.8	HIS584 (2.9), ASP1031 (2.7)	HIS584 (4.7), PRO551 (4.0), ILE552 (4.8), VAL555 (5.1), LEU1027 (5.2)
2‐(methoxymethyl)‐4‐oxopentanoic acid	−4.7	GLN369 (2.0), GLN369 (2.6), ARG370 (1.9), ARG370 (2.5), ARG370 (2.0), ASN1293 (2.0), GLY313 (3.4), ASN129 (3.5)	
Pyrazine, 2,5‐bis(1,1‐dimethylethyl)‐, 1,4‐dioxide	−5.8		TRP1297 (3.5), VAL587 (4.76), TRP1297 (4.4), VAL587 (5.2)
3,4‐Dihydro‐3,5,8‐trimethyl‐3‐(4,8,12‐trimethyltridecyl)‐(2H)1‐benzopyran‐6‐acetate	−7.1		VAL587 (4.9), VAL587 (4.1), MET1290 (5.3) LEU1149 (5.4), LEU592 (4.9), PHE591 (3.9), TYR1294 (4.8), TRP1297 (5.2), TRP1297 (4.3), VAL587 (4.7)
2,6‐Lutidine3,5‐dichloro‐4dodecylthio—	−5.6	ASN1293 (2.8), THR588 (2.4)	PRO551 (4.6), VAL587 (4.9), TRP1297 (3.6), TRP1297 (3.6)
alpha‐Tocopherol acetate	−7.6		TRP1297 (4.6), TRP1297 (3.7), LEU373 (5.2), ILE585 (5.08), PRO589 (4.6) TYR377 (4.9), TRP1297 (3.9), TRP1297 (4.3), TRP1297 (5.0), TRP1297 (4.6), TRP1297 (4.3), TRP1297 (4.9)
Cholesterol	−9.4		VAL587 (5.1), LEU1149 (4.8), VAL587 (3.8), LEU592 (4.2), LEU592 (4.0), HIS584 (4.9), HIS584 (4.5), PHE591 (4.7), PHE591 (4.5), TRP1297 (5.1), TRP1297 (4.2), TRP1297 (3.9), TRP1297 (4.4)
Stigmasterol	−9.8		TRP1297 (3.5), VAL587 (4.5), LEU592 (4.7), PRO551 (4.7), ILE552 (4.9), ILE552 (4.6), LEU1027 (4.1), LEU1027 (4.1), HIS584 (4.7), HIS584 (4.3), PHE591 (5.4), TRP1297 (4.7), TRP1297 (3.8), TRP1297 (3.9), TRP1297 (4.9), TRP1297 (4.1), TRP1297 (4.5)
beta‐Sitosterol	−10		TRP1297 (3.6), TRP1297 (3.6), LEU1149 (5.4), VAL587 (4.2), LEU592 (4.59), TYR377 (4.7), TYR1294 (4.8), TRP1297 (4.6), TRP1297 (3.9), TRP1297 (4.8)

## 4 Discussion

Postoperative pain management is frequently ineffective, with nearly 80% of patients reporting moderate to severe pain. New multimodal analgesia strategies use a combination of pain relievers to manage this pain effectively (Sinatra 2002).

NSAIDs, which target the COX enzyme, are essential for acute pain management. However, their use is limited due to side effects such as gastrointestinal bleeding, ulcers, kidney issues, and decreased platelet function (Varrassi et al. 2021).

The discovery of a second cyclooxygenase, COX‐2, led to the hypothesis that NSAID side effects could be reduced because inhibition of COX‐2 is more directly implicated in ameliorating inflammation, whereas inhibition of COX‐1 is related to adverse effects in the GI tract (Muhammad and Fatima 2015).

In this study, stigmasterol (−7.9 kcal/mol) had the highest binding affinity for the COX‐2 receptor (5KIR) in our investigation, followed by lanosterol (−7.8 kcal/mol).

Diclofenac had docking scores of (−6.7 kcal/mol), which is analogous to our compounds. As a result, in silico molecular docking confirmed MECG's analgesic efficacy.

*Cardyocalyx gyroids* have been shown to produce hypoglycemic effects in both normal and STZ‐induced diabetic rats. The study discovered that a 50 mg/kg dose of MECG extract significantly reduced early and late fasting plasma glucose levels compared to Glibenclamide, implying that higher doses or longer treatment times may be required for comparable effects.

The molecular mechanisms of phytochemicals from MECG were investigated using molecular docking against the T2D target SUR1 (6JB3). All phytochemicals exhibited high binding energy with the SUR1 agonist. Lanosterol (−10.3 kcal/mol), beta‐sitosterol (−10 kcal/mol), and stigmasterol (−9.8 kcal/mol) outperformed reference compounds like Glibenclamide (−9.4 kcal/mol) and gliclazide (−8.3 kcal/mol), indicating that these phytoconstituents could be promising hypoglycemic agents (Itou et al. 2022; Muhammad and Fatima 2015; Nikolić et al. 2022; H. Singh et al. 2017).

The purpose of the in silico analysis was to evaluate the inhibitory action of selected compounds through computational docking studies (E. Teoh 2016). To identify prospective MECG compounds for in silico research, we first assessed their suitability as therapeutic molecules.

Lipinski's rule of five is widely used in rational drug design to forecast drug similarity and drug‐likeness. All molecules meet Lipinski's rule criteria among selected compounds with available drug‐likeness data (Table 3). While our findings indicate that analgesics and hypoglycemics derived from MECG may be more effective than standard treatments, more research is needed to confirm their superiority over existing options. Furthermore, one limitation of our research is a lack of toxicity data for these phytoconstituents, as these experiments were conducted on mice, not in humans, which will be investigated in future studies.

## 5 Conclusion

Medicinal plants have been essential to traditional and ethnomedicine practices across many civilizations globally. Thus, selecting appropriate plant species, extracting the phytochemicals that provide particular therapeutic benefits, and optimizing these substances for improved effectiveness with minimum adverse effects are essential stages in this domain. To achieve these aims, we examined the methanolic extracts of *Codariocalyx gyroides* to evaluate their analgesic and hypoglycemic properties. Our in vivo investigations showed substantial antinociceptive and hypoglycemic effects. Additionally, we used in silico docking and ADME analysis to determine the possible bioactive compounds responsible for these effects. Our results demonstrate that Stigmasterol had a higher docking score (−7.9 kcal/mol) against cyclooxygenase‐2 (COX‐2) compared to diclofenac (−7.2 kcal/mol), suggesting a molecular explanation for the observed analgesic benefits. Lanosterol exhibited enhanced affinity (−10.3 kcal/mol) for the sulfonylurea receptor compared to the conventional hypoglycemic drug Glibenclamide (−9.2 kcal/mol). However, more research is necessary to confirm these compounds as viable prospects for development as analgesic and antidiabetic medications.

## Author Contributions

**Nilufar Sultana:** conceptualization (equal), project administration (equal), supervision (equal), visualization (equal), writing – review and editing (equal). **Mahmudul Hasan:** data curation (equal), formal analysis (equal). **Ishmam Ibnul Arabi:** methodology (equal), writing – review and editing (equal). **Zobayed Islam:** software (equal), writing – review and editing (equal). **Joana Julhash:** data curation (equal), formal analysis (equal). **Umme Hani:** writing – review and editing (equal). **Anika Sikder:** data curation (equal), formal analysis (equal), writing – original draft (equal). **Most Afroza Khanam:** data curation (equal), formal analysis (equal). **Abdullah Ripon:** writing – original draft (equal), writing – review and editing (equal).

## Ethics Statement

The Institutional Animal Ethics Committee of the Department of Pharmacy, School of Engineering, Science and Technology, Manarat International University, Bangladesh, authorized animal experiments used in the study. All authors declare that the “Principles of Laboratory Animal Care” (NIH publication No. 85‐23, revised 1985) and any relevant national regulations were followed. All experiments including the investigation of the plant under study were reviewed and authorized by the Manarat International University's ethical committee under (approval number MIU/SEST/ERC/2024002).

## Conflicts of Interest

The authors declare no conflicts of interest.

## Data Availability

All data and materials are contained and described within the manuscript. The data set was deposited in publicly available repositories. A voucher specimen (Collected by NS, accession no. DACB87278) has been preserved in the Bangladesh National Herbarium in Mirpur, Dhaka‐1216.
